# Loss of retinoic acid receptor-related receptor alpha (Rorα) promotes the progression of UV-induced cSCC

**DOI:** 10.1038/s41419-021-03525-x

**Published:** 2021-03-04

**Authors:** Guolong Zhang, Guorong Yan, Zhiliang Fu, Yuhao wu, Fei Wu, Zhe Zheng, Shan Fang, Ying Gao, Xunxia Bao, Yeqiang Liu, Xiuli Wang, Sibo Zhu

**Affiliations:** 1 Institute of Photomedicine, Shanghai Skin Disease Hospital, Tongji University School of Medicine, Shanghai, 200443 China; 2 State Key Laboratory of Genetic Engineering, School of Life Sciences, Fudan University, Shanghai, 200438 China; 3 Department of Epidemiology, School of Public Health, Fudan University, Shanghai, 200438 China; 4 Department of Pathology, Shanghai Skin Disease Hospital, Tongji University School of Medicine, Shanghai, 200443 China

**Keywords:** Squamous cell carcinoma, Oncogenesis

## Abstract

Cutaneous squamous cell carcinoma (cSCC) is prevalent in the world, accounting for a huge part of non-melanoma skin cancer. Most cSCCs are associated with a distinct pre-cancerous lesion, the actinic keratosis (AK). However, the progression trajectory from normal skin to AK and cSCC has not been fully demonstrated yet. To identify genes involved in this progression trajectory and possible therapeutic targets for cSCC, here we constructed a UV-induced cSCC mouse model covering the progression from normal skin to AK to cSCC, which mimicked the solar UV radiation perfectly using the solar-like ratio of UVA and UVB, firstly. Then, transcriptome analysis and a series of bioinformatics analyses and cell experiments proved that Rorα is a key transcript factor during cSCC progression. Rorα could downregulate the expressions of S100a9 and Sprr2f in cSCC cells, which can inhibit the proliferation and migration in cSCC cells, but not the normal keratinocyte. Finally, further animal experiments confirmed the inhibitory effect of cSCC growth by Rorα in vivo. Our findings showed that Rorα would serve as a potential novel target for cSCC, which will facilitate the treatment of cSCC in the future.

## Introduction

Cutaneous squamous cell carcinoma (cSCC) is the second most common non-melanoma skin cancer with 1.8 million incidences in a global context in 2017^1^. The progressions of cSCC from normal skin (NS) are determined by genetic susceptibility and environment risk factors including ultraviolet radiation, carcinogenic chemicals, and immunosuppressive drugs^2^. Although cSCC is characterized by a relatively low risk of metastasis and with long-term survival after treatment, there were still over 2.1% of cSCC patients that developed into lymphatic metastases and 1.5% deceased in 2012 in the US white population^3^. Furthermore, the ageing and growing population have led to an increasing incidence of cSCC, especially for the aged. Extensive researches have been carried out to uncover the pathogenesis behind cSCC from various omics aspects including genomics^4^, transcriptomics^5^, proteomics^6^, and epigenetics^7^.

Fourteen susceptive loci and 13 candidate genes including DEF8, IRF4, MC1R, etc. associated with high cSCC risk were identified by genome-wide association studies in different populations^8–10^. Except for the susceptive genes, high aberrated genes were also investigated in cSCC patients by whole-exome sequencing technology. In specific, NOTCH1/2, TP53, CDKN2A, HRAS, etc. held a very high mutated rate^11–14^. What is more, the expression profile of cSCC has been quantified by either gene expression chips or RNA-sequencing (RNA-seq) technology, and a large number of differentially expressed genes (DEGs) were detected^5,15^. As more and more focuses were received on the process of post-transcription and histone modification, the dissection of pathogenesis behind cSCC are spreading from genetics to epigenetics field^16,17^. However, although many significant features were identified enriched or suppressed in cSCC, the key factors underlying the progression of NS to cSCC remains to be elucidated.

Actinic keratoses (AK), histologically characterized by epidermal thickening and spinous layer atypia, is considered to be a precancerous lesion of cSCC^18^. The hazard of progression of AK to cSCC was estimated to be 0.6% within one year, then increased to 2.57% within 4 years^19^. Although, the estimated progression rate was low, ~65% cSCCs were diagnosed clinically as AKs, previously^19^. Thus, it is of great importance to uncover the mechanism involved in the progression progress from NS to AK, then to cSCC. On the contrary, it is more difficult to identify the driver gene than other malignancies, as cSCC is the most highly mutated malignancy with a complex genetic background^20^. Therefore, a functional-based biomarker is more essential to identify the mechanism. Lambert et al.^21^ found that the mitogen-activated protein kinase pathway may be important for the transition of AK to cSCC by comparing the transcriptome profiling of 10 AKs and 30 cSCCs. Similarly, Chitsazzadeh et al.^22^ detected a series of transcription factors (TFs) such as ETS2, SP1, FREAC2, and AP1 which should be responsible for the gene expression changes observed during the stage of AK and cSCC development by a cross-species identification strategy. However, the identified TFs either lacked necessary validation in terms of pathway or function nor explain the whole process from NS to cSCC via AK stage.

In the present study, we aim to identify transcriptomic markers playing critical roles in the progression of cSCC from NS and validate the AK as a transitional state. We firstly established a UVR-induced cSCC model using SKH-1 hairless mouse. Then, the transcriptome profiles among NS, AK, and cSCC tissues were investigated. We further constructed a pseudo-time trajectory to validate the NS-AK-cSCC path route, through which we found that loss of retinoic acid receptor-related receptor alpha (Rorα) was involved in the process. Further wet-lab assays and animal experiments were carried out to understand the function of Rorα and possible down-stream targets. This study unraveled the pathogenesis progression of UVR-induced cSCC, explained Rorα based function in the process, and enlighten a promising therapy of cSCC.

## Materials and methods

### UVR-induced cSCC mouse model construction and tissue collection

Eight 7-month-old female hairless SKH-1 mice purchased from Shanghai Laboratory Animal Center (Shanghai, China) were randomly and equally divided into two groups: ultraviolet free (UVF) and ultraviolet radiation (UVR) group. The UVR group was used to model UVR-induced cSCC under controlled conditions. In detail, mice in the UVR group were irradiated with ultraviolet rays from the SUV1000 daylight ultraviolet simulator (SIGMA, Shanghai, China) with built-in UVA and UVB filters. Preliminary experiments found that the initial minimal erythema dose (MED) was 160 mJ/cm^2^ for UVB and 2520 mJ/cm^2^ for UVA^23–25^. The UV irradiation was given four times every week for 28 weeks. As the mice in the UVR group have an increased tolerance to ultraviolet radiation as the experiment progresses by, the light dose needs to be enhanced till 1.625 MED for the end of 20 weeks. At the end of the experiment, the cumulative dose for UVA and UVB were 242.91 and 26.99 J/cm^2^, respectively. The tumor numbers and diameters in the UVR group were recorded weekly. After 28 weeks, all mice were euthanized by carbon dioxide inhalation.

**Fig. 1:**
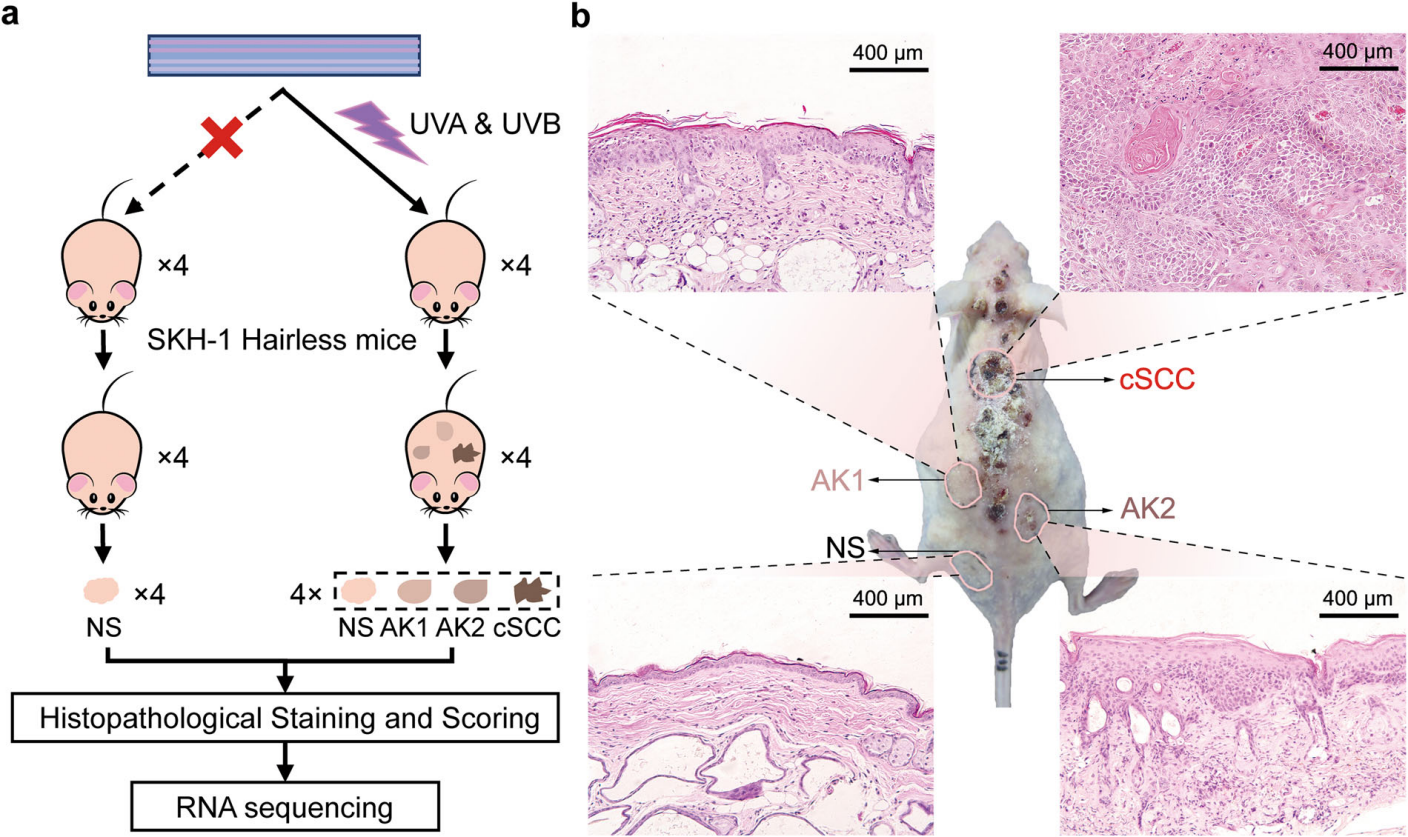
Establishment of UV-induced cSCC mouse model. **a** Diagram of experiment design of UV-radiated cSCC mouse. **b** Histology of representative sample of NS, AK-1/2 and cSCC from one UV- SKH-1 mouse. NS normal skin, AK actinic keratoses (AK1 and AK2 were grade 1 and grade 2 of AK), cSCC cutaneous squamous cell carcinoma.

All skin tissues were isolated from the back of mice (Fig. 1a). In specific, one piece of NS tissue was isolated from each mouse in the UVF group (NS-1, NS-2, NS-3, NS-4). In the UVR group, one piece of NS and three skin lesion tissues, including actinic keratosis grade 1 (AK-1), AK grade 2 (AK-2), and cSCC were collected. These tissues were validated by a pathologist with hematoxylin-eosin (HE) staining and defined as NS, AK-1, AK-2, and cSCC. For HE staining, part of each tissue was immediately fixed with 4% formaldehyde solution. For transcriptomic analyses, the rest part was collected in TRIZOL (Sigma, USA) solution for RNA extraction.

### Patient sample collection

Primary cSCCs and patient-matched normal adjacent samples for immunohistochemistry (IHC) staining were obtained from consecutive cSCC patients, all of whom were immunotherapy-naive. All diagnoses of cSCC were verified histologically by a board-certified dermatopathologist. The study was approved by the Institutional Review Board of Shanghai Skin Disease Hospital.

### mRNA library preparation for sequencing

Total RNA was extracted using TRIZOL reagent as per manufacturer’s instruction. cDNA libraries were prepared by Nextera sample preparation kit (Illumina, USA). Index labeled libraries sized at 300–450 bp length were recovered by using Ampure XP Beads (Beckman Coulter, USA). All libraries were quantified using 2100 Bioanalyzer and pooled as 1:1 at 2 nM for 150 paired-end modes and were further sequenced by NextSeq 500 (Illumina, USA).

### RNA-seq analysis

The quality of raw reads was evaluated by using FastQC (v0.11.5)^26^. Then, low-quality reads were filtered by Trimmomatic (v0.36)^27^. Reads with an average Phred quality score <20 were discarded after trimming. All the remaining qualified reads were aligned to the mouse reference genome (GRCm38) using HISAT2^28^. Then, StringTie was employed for gene expression quantification to build an expression matrix^29^.

Data pre-processing, *Pearson* correlation, and hierarchical clustering analysis calculated by ward method were performed with *base* and *stat* R-packages based on R software (v3.6.2). R-Packages including *limma*, *edgeR*, and *ggplot2*, *ComplexHeatmap* were used for data normalization and visualization. The identification of DEGs was performed by calculating fold changes (FC) and average expression differences using the normalized value of each gene, and statistical significance was tested by Student’s t-test. Then, DEGs were determined by the threshold of fold change >1.5 or <0.67, and *p* value < 0.05.

### Gene set variation analysis

In order to further identify pathways enriched in different groups, gene set variation analysis (GSVA) using gene set file of biological processes from the Molecular Signatures Database (c5.bp.v7.1.symbols.gmt, MSigDB v7.1 from http://bioinf.wehi.edu.au/software/MSigDB/) was performed as implemented in the *GSVA*^30^ R-package (v1.34.0, default parameters), in which the gene-by-sample matrix is converted into a pathway-by-sample matrix. Then, the differentially expressed pathway was identified by *limma* R-package with the same threshold in DEGs identification.

### Immune microenvironment analyses

In order to investigate the immune microenvironment (IME) change during cSCC progression, we next inferred the compositions of immune and stroma cells using enrichment score-based algorithm xCell from RNA-seq data^31^. Briefly, enrichment scores of 36 immune cells were obtained from the gene expression matrix of all 20 samples using *immunedeconv* R-package. Then, the T cell subset, monocyte, and neutrophil were generated by summation of the scores in each sample^32^. Heatmap was generated based on pathological stages. To further understand the relationship between the pathological stage and the IME change, we employed univariate regression analysis between TPS (tumor progression stage) and cell subsets fraction.

### Pseudo-time trajectory construction

To investigate the progression trajectory of cSCC, we used minimal spanning tree algorithm-based *Monocle* (v2) R-package to perform a pseudo-time analysis. We assumed that the trajectory had a tree structure, with a root state of related NS, and a leave state of tumor cells. Samples were colored as their pathological classifications.

### Transcription factor co-regulatory network

Gene co-expression networks were firstly built according to the normalized gene expression values. The *Pearson* correlation coefficients of genes were obtained as the gene-to-gene co-expression adjacency matrix. We further applied transcription factors as core genes (AnimalTFDB, v3.0)^33^ and removed nodes with absolute correlation coefficient <0.5, false discovery rate >0.05 or number of edges <5, to draw a transcription factor co-regulatory network CytoScape (v3.7.3).

### External validation of Rorα expression and survival analysis

Rorα expression was investigated by immunochemistry staining and single-cell datasets. The pathology-stained micrographs of Rorα from normal and cSCC tissues were downloaded from the Human Protein Atlas (HPA) database. The optical density (OD) of Rorα between NS tissue and cSCC pathological was compared by ‘Image J’ software. Furthermore, large-scale single-cell RNA-sequencing (scRNA-seq) data from human cSCCs and matched NS with 48,164 cells (GSE144240) was obtained from Gene Expression Omnibus (GEO) database, which enabled us to analyze the Rorα expression at single-keratinocyte level. As cSCC database with clinic information was unavailable and head and neck squamous cell carcinoma (HNSCC) is often used as an alternative of cSCC in most studies^22,34^. Thus, we used HNSCC to explore the association between Rorα and survival. TCGA based survival data were downloaded from OncoLnc (http://www.oncolnc.org/). The analysis was performed with *survival* and *survminer* R-packages.

### IHC staining of cSCC patients

cSCCs and patient-matched normal adjacent samples were fixed in 10% neutral buffered formalin, dehydrated with gradient ethanol, hyalinized in xylene, embedded in paraffin, and made into sections. Sections (5 µm) of paraffin-embedded tissue were deparaffinized, dehydrated with gradient ethanol, and rinsed with tap water before antigen repair and serum blocking. Next, the primary anti- Rorα antibody (Abcam: ab60134, 0.5–10 μg/ml) was added for overnight reaction at 4 °C, followed by the addition of secondary antibody for 1 h of incubation. 3, 3′-Diaminobenzidine tetrahydrochloride solution was used for 15 min of coloration, followed by counterstaining with hematoxylin, dehydration with gradient ethanol, hyalinization with xylene, mounting with neutral resin, and observation under a microscope.

### Interference, activation assay, and qRT-PCR of RORA, S100a9, and Sprr2f

Human-derived cell line A431, HaCaT, and mouse cell line PECA from the National Infrastructure of Cell Line Resource were seeded in cell culture dishes and cultured for 3 days till confluency at 90%. Since Rorα expressed lowly in cancer cells (data not shown), we employed Rorα agonist only to understand its function. Rorα agonist SR1078 (Selleck, USA) and small interfering RNA (siRNA)-Hilymax complex (S100a9 and Sprr2f) were added to the cells, incubated at 37 °C for 48 h.

After reverse transcription of RNAs from cells, a quantitative real-time PCR (qRT-PCR) reaction system was setup. SYBR green dye (Takara) was applied in the PCR reaction. The Ct values of the target genes S100a9, Sprr2f, and the housekeeping gene GAPDH were obtained, and the 2^−ΔΔ^Ct algorithm was used for quantitative analysis. Primer and siRNA information were presented in Table 1.

**Table 1:** Primer and siRNA information. Design of qRT-PCR primers and siRNAs.

Primer	Sequence (5′-3′)
Homo-S100a9-F	AACATAGAGACCATCATCAACACC
Homo-S100a9-R	GGTCCTCCATGATGTGTTCTATG
Mouse-S100a9-F	TCAATACTCTAGGAAGGAAGGACA
Mouse-S100a9-R	AGCTGATTGTCCTGGTTTGT
Homo-Sprr2f-F	CCTGCCCACCATCAAAGT
Homo-Sprr2f-R	CTTGCTCTTGGGTGGACA
Mouse-Sprr2f-F	TGAGCCTTGTCCTCCTCCA
Mouse-Sprr2f-R	TCTTGGGTGGGCACTTCTG
Homo-GAPDH-F	GAGTCCACTGGCGTCTTCAC
Homo-GAPDH-R	ATCTTGAGGCTGTTGTCATACTTCT
Mouse-GAPDH-F	TGACCTCAACTACATGGTCTACA
Mouse-GAPDH-R	CTTCCCATTCTCGGCCTTG
Sprr2f-215-siRNA sense	GCAGAAAUGUCCUCCUGUGTT
Sprr2f-215-siRNA antisense	CACAGGAGGACAUUUCUGCTT
S100a9-267-siRNA sense	GCUUCGAGGAGUUCAUCAUTT
S100a9-267-siRNA antisense	AUGAUGAACUCCUCGAAGCTT

*F* forward, *R* reverse, *si* small interfering.

### CCK-8 assay, wound-healing assay, transwell assay, and apoptosis assay

For Cell Counting Kit-8 (CCK-8) (Dojindo, Japan) assay, after cells were activated or interfered for 24 and 48 h, 10 μl CCK-8 was added to each well. OD value of each well was measured at 450 nm using a microplate reader.

For the wound-healing assay, after the cells reached 90% confluency, a pipette tip was used to scratch a line on the cell layer. The picture of each wound was recorded by microscope after 0 and 24 h. Image J software was used to calculate the mean value of the distance of each wound.

For transwell assay, cells were seed at upper microholed wells with Matrigel plated containing serum-free RPMI 1640 medium. The wells were placed into the lower well containing 500 µl of complete medium (RPMI 1640 and 10% fetal bovine serum). After incubation at 37 °C for 48 h, cells were gently removed in the upper wells with a cotton swab. The cells in the lower chamber were fixed with 5% glutaraldehyde for 10 min and stained with 1% crystal violet dye in 2% ethanol at room temperature for 20 min, then photographed and counted by Image J.

For apoptosis assay, cells were resuspended cells in fixation buffer and stained with Annexin V-FITC and Propidium Iodide solutions respectively for 30 min on ice. Then cells were then analyzed by flow cytometry (BD Biosciences, USA) with FITC and PI channel.

### Animal experiment

To investigate the effect of SR1078 on cSCC growth in vivo, we conducted an animal experiment. Firstly, 10 eight-week-old SKH-1 mice were randomly divided into two groups (*n* = 5 per group). The injected dose of SR1078 was 20 mg/kg, and the weight of each mouse was estimated to an average of 25 g. SR1078 was dissolved in the solution including 10% dimethyl sulfoxide (DMSO, Selleck), 40% PEG300 (Selleck), 5% Tween80 (Selleck) and 45% ddH_2_O. The agonist group and control group were injected intraperitoneally with 150 µl SR1078 solution and DMSO solution, respectively. In specific, three times injection in a week was performed before cSCC cell inoculation. Then, XL50 cSCC cells from China Center for Type Culture Collection (Wuhan, China, 5 × 10^6^) and NIH/3T3 cells (mouse embryonic fibroblast, 1 × 10^6^) were injected subcutaneously into the backs of mice to establish a cSCC mouse model. After incubation, mice were injected intraperitoneally with SR1078 or DMSO solution daily. Tumor volume was measured every 2 days and calculated as length × width^2^ × 0.5 (mm^3^). Eighteen days post-injection, the tumor was harvested for cell proliferation evaluation by IHC of Ki67 expression (MA5-14520, Thermo Fisher Scientific) following the guideline.

## Results

### Mouse model and samples collection

In order to simulate sun exposure accurately, we employed solar simulators containing UVA and UVB to establish cSCC mouse model. Eight SHK-1 Hairless mice were used for the present study, among which four mice were in the UVF group and four were in the UVR group (Fig. 1a). Four NS tissue samples were isolated from the UVF group. After mice developed into cSCC, cSCC tumor samples and matched NS and AK (grade 1 (AK1) and grade 2 (AK2)) samples were isolated from the UVR group (Fig. 1a). The pathological classification and the tumor pathological scoring (TPS) of tissues were also presented (Table 2). All experiment samples were histologically validated. Representative samples regarding NS, AK1, AK2, and cSCC were histologically imaged (Fig. 1b). NS samples showed a uniform distribution of keratinocytes, while AK1 and AK2 exhibited a histological phenotype of UV damage including elastosis, fibrosis, and thicker epidermis. Compared with the former, the tumor cells in cSCC distributed in nests with large deeply staining nuclei, and also with many karyokinetic and acantholytic cells.

**Table 2:** Tumor pathological classification and scoring of mice skin tissues.

Groups	Subject	Sample name	Pathology	Pathological scoring (0–5)
Ultraviolet free (UVF)	1	NC-1	NS-1	0
	2	NC-2	NS-1	0
	3	NC-3	NS-1	0
	4	NC-4	NS-1	0
Ultraviolet radiation (UVR)	5	1-NC	NS-2	0
		1-AK-1	Grade 1 papilloma (G1P)	1
		1-AK-2	Grade 2 papilloma (G2P)	2
		1-cSCC	cSCC	4
	6	2-NC	NS-2	0
		2-AK-1	Grade 1 papilloma (G1P)	1
		2-AK-2	Grade 2 papilloma (G2P)	2
		2-cSCC	cSCC	5
	7	3-NC	NS-2	0
		3-AK-1	Grade 1 papilloma (G1P)	1
		3-AK-2	Grade 3 papilloma (G3P)	3
		3-cSCC	cSCC	4
	8	4-NC	NS-2	0
		4-AK-1	Grade 1 papilloma (G1P)	1
		4-AK-2	Grade 3 papilloma (G3P)	3
		4-cSCC	cSCC	4

### Differences among NS, AK, and cSCC

**Fig. 2:**
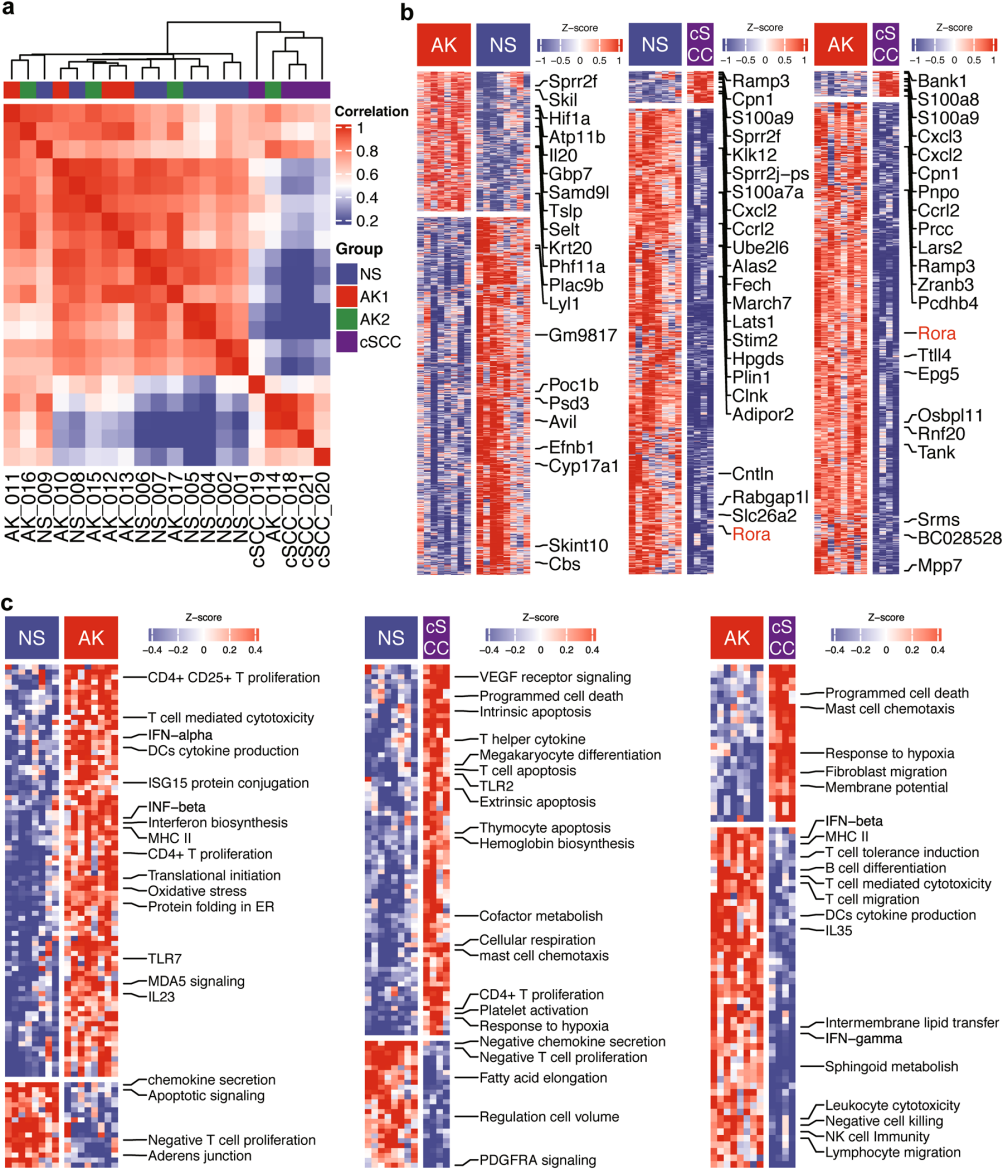
transcriptome overview across normal and UV-induced AK and cSCC development. **a** Unsupervised clustering using all genes analysis showed that cSCC were significantly different compared with AK and NS. **b** DEGs among each comparison. (**c**) GSVA analysis among each comparison. The comparisons include AK vs. NS, cSCC vs NS, and cSCC vs. AK.

RNA-seq was performed on NextSeq 500 with a total of 70 million reads generated. Unsupervised hierarchical clustering analysis using all genes showed that the NS and AK were closer compared to the cSCC cluster, showing a transcriptomic similarity over the NS and AK groups (Fig. 2a). A similar pattern was also observed in the principal component analysis (PCA) using all genes (Fig. S1). Specifically, there was no clearly different gene expression between AK1 and AK2. After performing DEGs identification, a total of 2022, 2031, and 1617 DEGs were identified in the comparisons of AK vs. NS, cSCC vs. NS, and cSCC vs. AK, respectively. The top 10 significantly up- and downregulated genes were highlighted (Fig. 2b). In general, genes such as S100 family, Sprr family, Cxcl family, Ccl family, and Mmp family members were significantly upregulated in cSCC (Fig. 2b).

Next, we used GSVA approach to identified differentially expressed pathways among NS, AK, and cSCC based on gene set file of biological processes from the Molecular Signatures Database. A total of 70, 67, and 76 differentially expressed pathways were identified among each comparison (Fig. 2c). Compared with NS, CD4^+^/CD25^+^ T proliferation, IFN-α, IFN-β, TLR7, and IL23 pathways were significantly upregulated, while chemokine secretion, Apoptotic signaling, and negatively modulate T cell proliferation pathways were upregulated in AK. In the comparison between cSCC and NS, VEGF receptor signaling, mast cell chemotaxis, response to hypoxia, megakaryocyte differentiation TLR2, and T cell apoptosis pathways were significantly increased. Finally, regarding the comparison between cSCC and AK, the increased pathways of programmed cell death, mast cell chemotaxis, response to hypoxia and fibroblast migration were observed, and IFN-β, IFN-γ, B cell differentiation, and lymphocyte migration pathways and etc. were downregulated in cSCC (Fig. 2c).

As most differentially expressed pathways were immune-related, we next sought to observe the change of immune microenvironment across NS, AK, and cSCC by RNA-seq deconvolution. The heatmap of the relative expression of immune cells is shown in Fig. S2a. Endothelial cells, cancer-associated fibroblast, natural killer, and CD4^+^ T helper1 (Th1) were hyperactivated in the cSCC group. Regulatory T cells and Granulocyte-monocyte progenitor hold the highest expression in the AK group. The highest microenvironment score and stroma score were observed in cSCC group (Fig. S2b). The immune cells composition regarding sample types were presented in Fig. S2c, d, e. The regression analysis showed that endothelial cell and neutrophil are positively correlated with TPS, indicating an extensive increase of angiogenesis and neutrophil accumulation during cSCC progression (Fig. S2f, g).

### Rorα is inhibited in cSCC

**Fig. 3:**
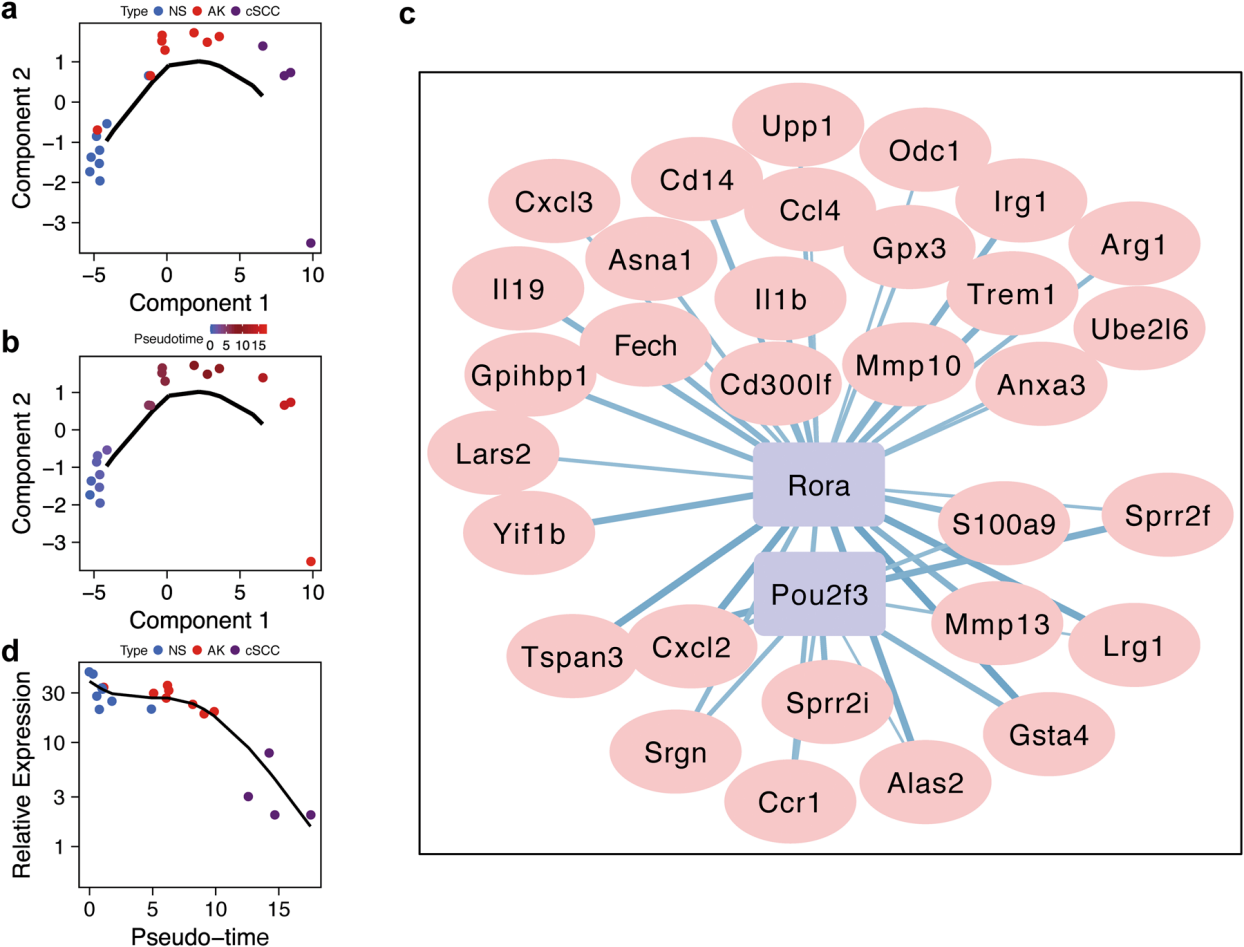
Reconstructed progression trajectory of UV-induced cSCC are regulated by Rorα. **a** Distribution of NS, AK, and cSCC samples in the pseudo-time trajectory analysis. **b** Samples were highlighted by pseudo-time in the trajectory, which showed a progression from NS to AK then cSCC. **c** Co-expressed network calculated between TFs and genes among the cSCC progression-driven genes. Purple rectangle nodes represent TFs and plink elliptic nodes represent genes. **d** Rorα expression with regards to the trajectory pseudo-time. Rorα expression was significantly downregulated in cSCC group.

The cSCC model was generated by irradiation of UVR from NS. The undetermined tumorigenesis process is intriguing and prompts us to further explore the progression trajectory. The gene expression profile of different stages allowed us to reconstruct the trajectory using. The graph showed a linear progression trajectory, which was fitted to the UVR-induced pathology stage very well (Fig. 3a). The solid black line represented the main route of the minimal spanning tree constructed, which exhibited the backbone and order of the cSCC development along a pseudo-temporal continuum. The trajectory clearly showed the tree starts from NS, goes through AK and ends with cSCCs (Fig. 3b). Then, we further constructed a co-expressed regulatory network between TFs and DEGs, which was involved in the trajectory. The TFs, including Rorα and POU domain, class 2, transcription factor 3 (Pou2f3) were found to be involved in the cSCC progression, which co-expressed with S100a9 (a member from the S100 family) and Sprr2f (a member from the Sprr family) (Fig. 3c). In particular, S100a9 and Sprr2f were significantly upregulated in cSCC tissues from the mouse model (*p* < 0.05, Fig. S3). Compared with NS, Sprr2f were also significantly upregulated in AK (*p* < 0.05), but no significant up-regulation of S100a9 was observed in AK. In the trajectory of NS-AK-cSCC, the expression of Rorα showed a continual decreasing trend in terms of the pathology stage, especially in cSCC (Fig. 3d).

**Fig. 4:**
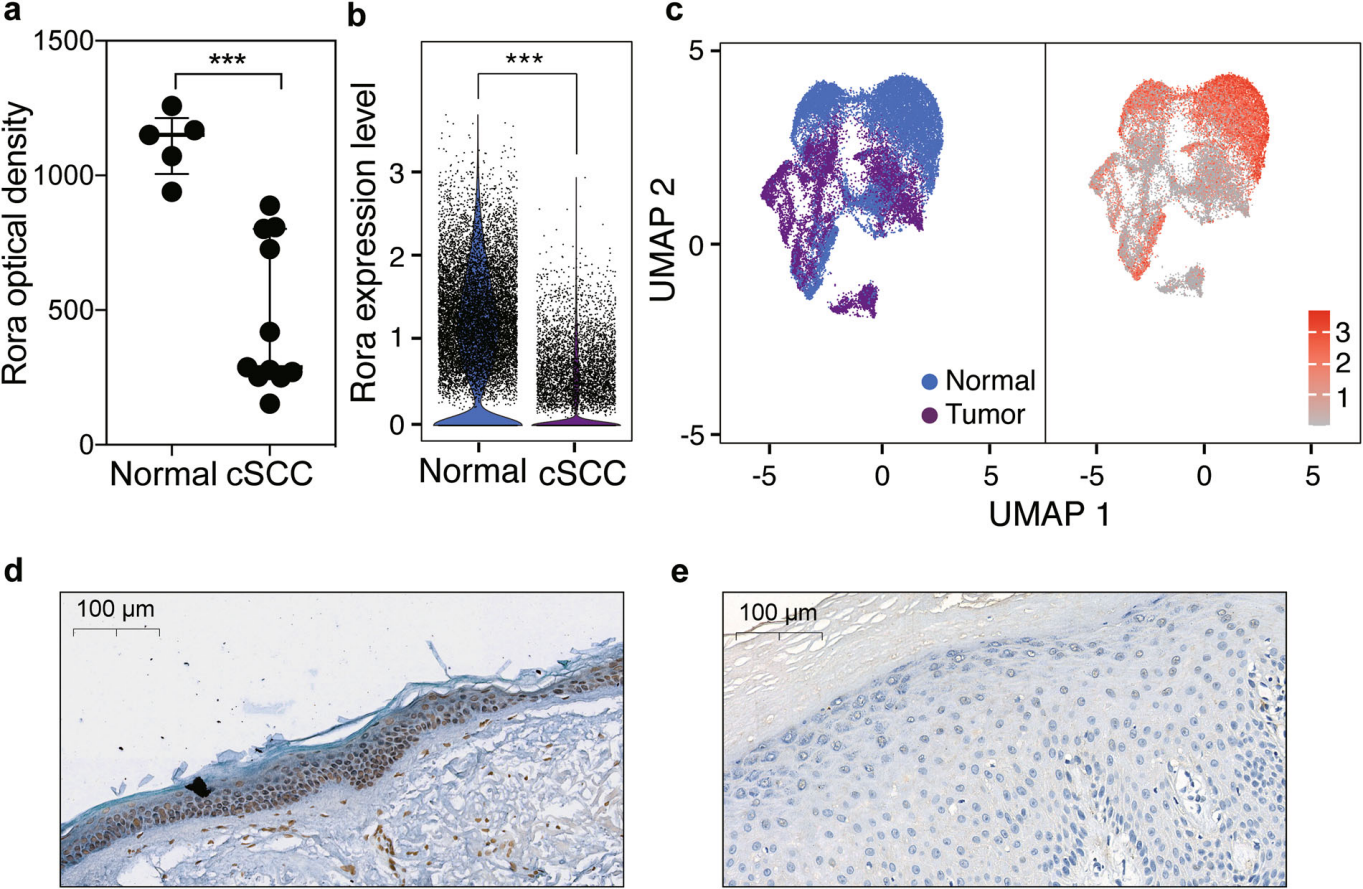
Rorα was downregulated in cSCC and associated with survival time of patients. **a** Optical density of Rorα. Data were extracted from HPA database and lower optical density was obtained in cSCC. **b** Expression of Rorα from single-cell RNA-sequencing data. Rorα was significantly downregulated in cSCC cells than that of normal keratinocytes. **c** Cells were highlighted by disease state and Rorα expression level by uniform manifold approximation and projection (UMAP). **d**, **e** Representative micrographs of Rorα expression by IHC in humans. Rorα in the normal tissue showed a significantly higher expression than that in the cSCC tissue. ***p < 0.001.

In order to further validate this result, we re-analyzed the histological staining of Rorα between NS tissue (five samples) and cSCC tissue (fifteen samples) from the Human Protein Atlas database. A lower relative density of Rorα was observed in cSCC (*p* < 0.01, Fig. 4a), indicating that the expression level of Rorα in cSCC is significantly lower than that of NS, which is in accordance with our result. In addition, as a recent cSCC-related single-cell transcriptomic data with 48,164 cells was released (GSE144240), we further investigate the Rorα expression of skin epithelial cells, from healthy and cSCC tissues. As the violin chart showed, Rorα was significantly downregulated in cSCC keratinocytes. (Fig. 4b, c). Furthermore, we calculated the percentage of cells with Rorα > 1 count, that more Rorα positive cells were exhibited in normal (72.49%) keratinocytes than that in the tumor (33.15%) cells. In summary, we solidly showed that Rorα was significantly downregulated in cSCC, which was inhibited during cSCC progression.

IHC staining was performed on the cSCC tissue and NS tissue in cSCC patients. The results showed that Rorα highly expressed in the nucleus of keratinocytes in NS, while the expression level in cSCC tissues is significantly reduced (Fig. 4d, e). In order to examine the effect of Rorα on survival time, we employed HNSCC data, which was similar to cSCC. The top 25% of high and low Rorα expression level patients were used for generating a 5-year Kaplan-Meier survival curve. It was found that the patients with high expression of Rorα achieved a better survival prognosis (*p* < 0.05, Fig. S4). To conclude, we validated the lower expression of Rorα in both human cSCC, and the survival results suggested that Rorα plays an important role for patients' survive.

### Rorα activation inhibits cSCC cell proliferation and migration

**Fig. 5:**
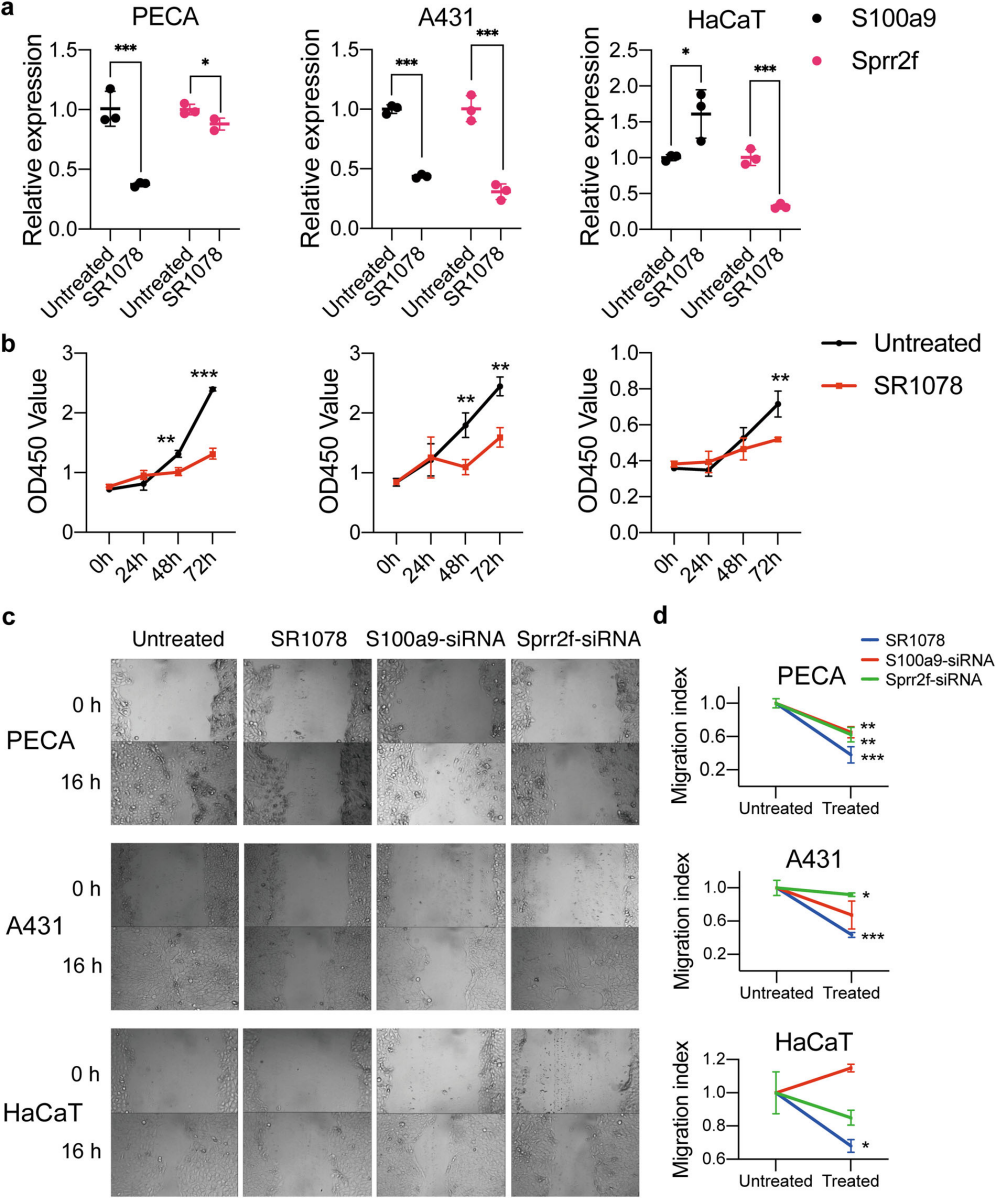
Progression of mouse and human cSCC cell lines were inhibited by Rorα activation in terms of invasiveness and proliferation. **a** Relative expression of S100a9 and Sprr2f expression after treated with Rorα agonist (SR1078) in skin carcinoma cells of PECA, A431 and normal keratinocytes of HaCaT, respectively. **b** CCK-8 assay showed that Rorα could inhibit the proliferation of PECA, A431 and HaCaT. **c**, **d** Rorα has an obvious inhibitory effect on cell migration, similarly, S100a9 and Sprr2f show the same trend in PECA and A431 cells. All asterisk marks denote the labeled samples are compared with the untreated group. *p < 0.05; **p < 0.01, ***p < 0.001.

To evaluate the role of Rorα in terms of the proliferation and migration ability we performed mouse and human cell-line based assays. As S100a9 and Sprr2f were predicted as genes that are regulated by Rorα, we further investigated the expression of these two genes by giving Rorα activator in cell lines. In general, S100a9 and Sprr2f were potentially downregulated in cSCC cells (PECA and A431) after being treated with Rorα agonist SR1078. S100a9 was downregulated in both PECA and A431 cells (*p* < 0.001), while extremely downregulation of Sprr2f was observed in A431 and HaCaT cells (*p* < 0.001). In the meanwhile, S100a9 expression was slightly upregulated in the HaCaT cell (*p* < 0.05) (Fig. 5a). Rorα agonist inhibited cell proliferation after stimulating all three types of cells (HaCaT: 72 h, *p* < 0.01; A431: 48 h, *p* < 0.01; 72 h, *p* < 0.01; PECA: 48 h, *p* < 0.01, 72 h, *p* < 0.001; Fig. 5b). By using siRNA interference with S100a9 and Sprr2f, no proliferation alteration of all three cells was seen (Fig. S5). In the wound-healing assay, all three cell lines showed significant migration inhibition after being stimulated by Rorα agonists (PECA: *p* < 0.01; A431: *p* < 0.01; HaCaT: *p* < 0.05). The cell migration of PECA (*P* < 0.05) and A431 (*p* < 0.05) was inhibited after siRNA interfered with S100a9, while there was a significant inhibitory effect after siRNA interferes with Sprr2f in PECA cell (*p* < 0.01) (Fig. 5c, d). There was no effect on cell remote migration ability after up and downregulated the expressions of these three genes in terms of the transwell assay (Fig. S6). Finally, we employed fluorescent flow cytometry to reveal that the changed function of these three genes did not affect the apoptosis in both keratinocytes and cSCC cells (Fig. S7). In conclusion, activation of Rorα could downregulate the expressions of S100a9 and Sprr2f in cSCC cells, which play important role in cSCC progression. Furthermore, Rorα can inhibit cell proliferation and migration in cSCC cells, but not the normal keratinocyte.

### Rorα activation inhibits cSCC growth

**Fig. 6:**
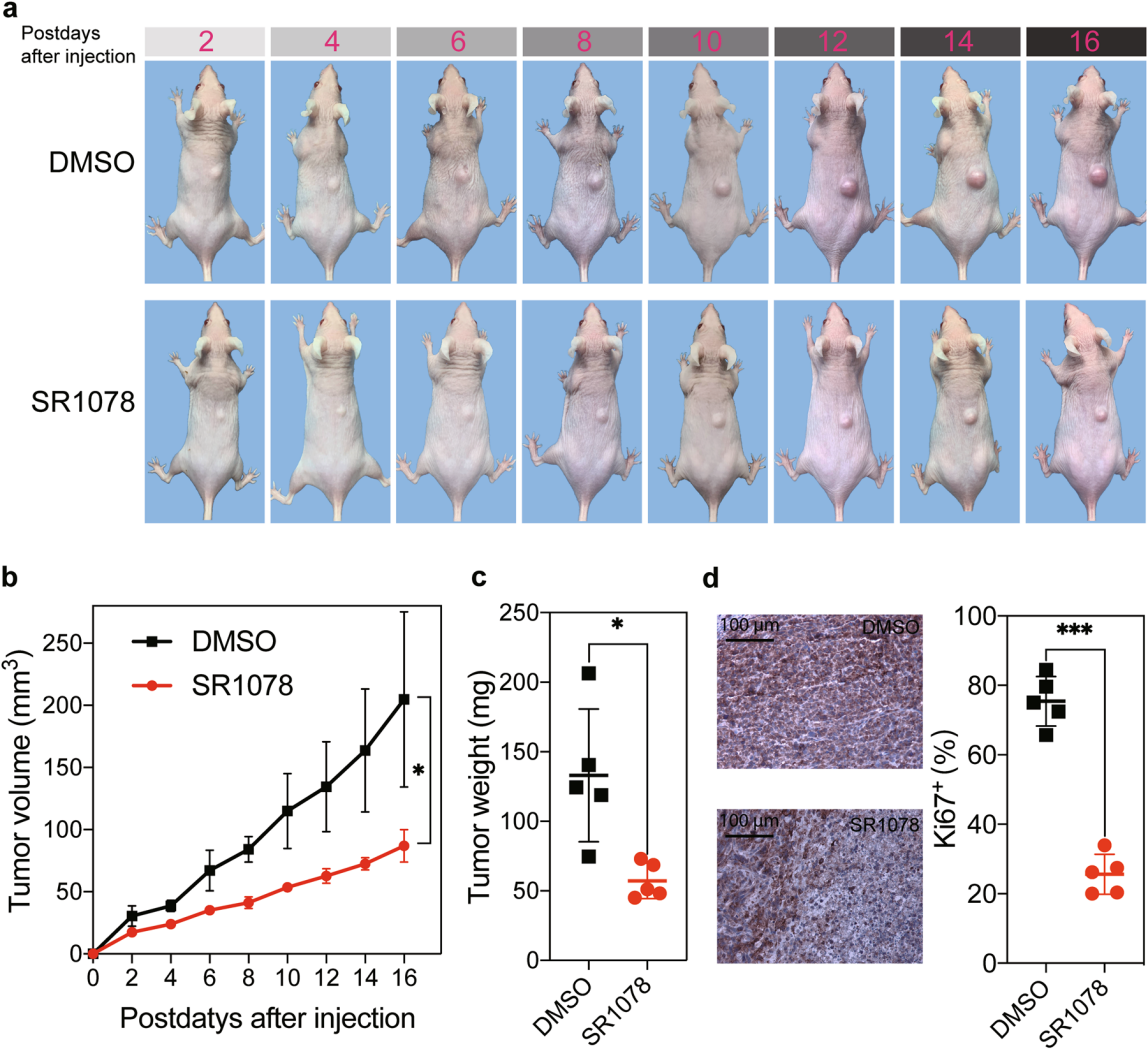
Rorα activation inhibits cSCC growth. **a** Representative tumors growth of SKH-1 mice after injected with cSCC cells between DMSO and SR1078 groups. **b** Tumor growth curve measured by tumor volume for DMSO and SR1078 groups. Tumor volumes were measured every two days and calculated as length × width^2^ × 0.5 (mm^3^). **c** Tumor weight at sixteen days after injection with cSCC cells. Data are presented as the means ± standard deviation. **d** Ki67 expression by IHC for DMSO and SR1078 groups. The proliferation of cSCC cells was attenuated by Rorα activation. *p < 0.05; **p < 0.01, ***p < 0.001.

To investigate whether Rorα could affect the cSCC growth in vivo, we constructed a cSCC model by injecting XL50 cSCC cells subcutaneously into SKH-1 mice. Overexpressing Rorα showed an inhibitory effect on tumorigenesis (Fig. 6a). Tumors in mice treated with Rorα agonist were significantly smaller than those of control mice (mean tumor volume: 86.82 vs. 204.77 mm^3^; *p* < 0.05; Fig. 6b). Then, tumors were weighed at the end of the experiment. Similarly, significantly lighter tumors were observed in the Rorα agonist group than the control group (57.28 vs. 132.96 mg; *p* < 0.05; Fig. 6c). Finally, the isolated tumors were used to evaluate cell proliferation by IHC of Ki67 expression between SR1078 and the control group. The result showed that the proliferation of cSCC cells was attenuated by Rorα activation (Fig. 6d). Overall, Rorα is a critical regulator of tumor growth in cSCC, and the activation of Rorα can inhibit its growth.

## Discussion

cSCC is quite common in melanoma skin cancer, and UV exposure including UVA and UVB was considered as a main pathogenic factor. by overwhelming epidemiological, clinical, and biological data, it was thought that UV could cause considerable accumulated DNA damage and has been classified as a category 1a carcinogen by International Agency for the Research on Cancer (IARC)^35^. Study has demonstrated that there were many differences between UVA- and UVB-regulated miRNAs in human primary keratinocytes^36^, which indicates the unique detrimental function of both UVA and UVB. A recent study generated a cSCC mouse model only using UVB irradiation, and the UVB-regulated or the combination of UVA- and UVB-regulated pathways involved in the cSCC progression may be ignored^22^. In the present study, we constructed a UV-induced cSCC mouse model covering the progression from NS to AK to cSCC, which mimicked the solar UV radiation perfectly using the solar-like ratio of UVA and UVB. The pathology of each stage was confirmed histologically.

A large number of DEGs were identified among NS, AK (the preneoplastic intermediate), and cSCC. Analyzing the complete gene expression data showed that AK held a similar mode with NS, regardless of the AK grades (Fig. 2a). This result was different from the study of Chitsazzadeh et al.^22^ that AK is more similar to cSCC with respect to the unsupervised clustering. This could be explained by the use of only UVB rather than the combination of UVA and UVB. Also, they only used the identified DEGs to generate the heatmap, which would further affect the clustering result. Although AK is similar to NS at a whole-transcriptome level, many identical DEGs were detected in AK and cSCC when compared with NS. Matrix metalloproteinase (Mmp) related genes including Mmp10 (stromelysin-2) and Mmp13 (collagenase-3) were detected in both AK and cSCC, compared with NS. Moreover, Mmp8 and Mmp9 were also upregulated in cSCC. In specific, Mmp13 showed the highest fold change among Mmps in both AK and cSCC. It has been reported that Mmp10 and Mmp13 are highly expressed in tumor cells in cSCC^37,38^, and almost universally upregulated across all cancers by TCGA database analysis^39^. The up-regulation of Mmps will degrade the extracellular matrix, which will promote cSCC cell invasion and implantation. Furthermore, the up-regulation of Mmps in AK implies that it may also promote the progression of AK to cSCC.

Chemokines are essential coordinators for cell migration and interactions, which have a great impact on tumor development. Many chemokines were identified in cSCC and AK. In cSCC, Cxcl2 and Cxcl3 were identified, showing an angiogenic effect^40^. However, Cxcl9 and Cxcl10 were identified in AK, which exhibit an angiostatic effect^40^. These results were determined by the malignant biological behavior in cSCC. When a mass of tumor cells struggle to rapidly proliferate, the tumor system requires accelerated neoangiogenesis to obtain more oxygen and nutrient, while no extra oxygen is required by AK. Inflammation is important in cancer. In the present study, inflammation-related chemokines including CCL3 and CCL4 were both increased in cSCC and AK, which helps recruit and CD4^+^, CD8^+^ T cell to kill tumor cells^41^. A similar result was confirmed by GSVA analysis (Fig. 2b). Pathways of VEGF receptor signaling, hemoglobin biosynthesis, response to hypoxia, cofactor metabolism and mast cell chemotaxis and *etc*. were significantly un-regulated in cSCC.

Although most studies considered that AK is a kind of precancerous lesion of cSCC, little study confirm the result at a transcriptome level. In the present study, a linear trajectory was re-constructed by transcriptome data. The progression trajectory clearly demonstrated that AK can progress into cSCC. From the genes involved in the pseudo-time trajectory, two TFs called Rorα together with Pou2f3 were sought out by co-expression analysis, which are regulatory factors for many genes including S100 family and Sprr family. Overexpression of Pou2f3 significantly inhibited the proliferation of HaCaT cells at 48 and 72 h after transfection^42^. In addition, Pou2f3 has been suggested as a tumor suppressor gene, which was downregulated in cervical cancer and melanoma^43,44^. Retinoid orphan nuclear receptors (RORs) are subfamily of the orphan nuclear receptors, which have been demonstrated to play vital roles in tumor progression and to be attractive therapeutic targets for many cancers^45,46^. Rorα is one of the members of ROR subfamily and downregulated in many malignancies. In the present study, Rorα was predominantly downregulated in cSCC (Fig. 3d), and the low expression of Rorα was confirmed by both scRNA-seq and solid biological experiments. We found that activation of Rorα inhibited cSCC cell proliferation and migration. In addition, we confirmed the anti-tumor effect of Rorα activation in a cSCC mouse model in vivo. Extensively low expression of Rorα was noted in sixteen kind of cancers^47^. Furthermore, Rorα was proved to be responsible for the normal differentiation of keratinocyte, and showed a low expression in cSCC^48^. A positive correlation was also observed between the low expression of Rorα and melanoma progression and short overall survival rate^49,50^, as well as unfavorable prognosis of breast cancer^51^, which can directly downregulate tumor suppressor gene Semaphorin 3F (Sema3f), causing tumor progression^52^. The extensively low expression of Rorα indicates an essential role in tumor progression.

Recent studies have suggested that the changed expression of S100 family may play a key step during cancer development and frequently observed in human epithelial tumors^53^. Here, we also noted that S100a9 was regulated by Rorα and upregulated in cSCC (Fig. 3c). After being treated with Rorα agonist in human and mouse cSCC cells, S100a9 expression was significantly downregulated, which confirmed the regulation effect of Rorα. The further cell experiment confirmed the effect of proliferation and migration in cSCC cells by S100a9. S100a9 was also found to be upregulated in breast, lung, gastric, colorectal, pancreatic, and prostate cancer^54^, while downregulated in squamous oesophageal carcinomas^55^. Compared with extensive reports of S100a9, litter studies were published about Sprr2f. Sprr2f is a cross-linked envelope protein of keratinocyte, that first appears in the cell cytosol, but ultimately becomes cross-linked to membrane proteins by transglutaminase. Uniport data base showed that it is associated with cornification, epidermis development (https://www.uniprot.org/), the same function was proved by Takatori et al.^56^. A recent in silico analysis showed that Sprr2f was associated with ovarian carcinoma^57^, which indicates that further study showed be conducted to elucidate the specific function of Sprr2f in tumor.

## Conclusion

In summary, Rorα and Pou2f3 were identified to be significantly downregulated in cSCC by a UVR-induced cSCC mouse mode, which perfectly mimics the sunlight radiation in daily life. Solid bioinformatics analyses including scRNA-seq data and cell experiment together validated the expression of Rorα. Moreover, the cell experiment confirmed the inhibition effect in cell proliferation and migration of Rorα by downregulating S100a9 and Sprr2f. Further anti-tumor effect of Rorα activation was confirmed in a cSCC mouse model in vivo. Our findings demonstrated that Rorα would serve as a potential novel target for cSCC, which will facilitate the treatment of cSCC in the future.

## Supplementary information

Supplementary

## Data Availability

Raw data sets related to this study are available through the Gene Expression Omnibus (GSE158634).
