# Supplementary material for: Loss of retinoic acid receptor-related receptor alpha (Rorα) promotes the progression of UV-induced cSCC
Source: Cell Death Dis. 2021 Mar 4;12(3):247. doi: 10.1038/s41419-021-03525-x (PMC7933246; doi:10.1038/s41419-021-03525-x)
Supplement: Supplementary file 1 — Supplementary [file 41419_2021_3525_MOESM1_ESM.docx]

Supplementary Materials

Loss of retinoic acid receptor-related receptor alpha (Rorα) promotes the progression of UV-induced cSCC

Guolong Zhang ^1,†^, Guorong Yan ^1,†^, Zhiliang Fu ^2^, Yuhao wu ^1^, Fei Wu ^3^, Zhe Zheng ^1^, Shan Fang ^1^, Ying Gao ^1^, Xunxia Bao ^2^, Yeqiang Liu ^3,*^, Xiuli Wang ^1,*^ and Sibo Zhu ^2,*^

^1^ Institute of Photomedicine, Shanghai Skin Disease Hospital, Tongji University School of Medicine, Shanghai, 200443, China; zglamu@163.com (G.Z.); guorongyan@tongji.edu.cn (G.Y.); wuyuhao@tongji.edu.cn (Y.W.); zhengzhe16@163.com (Z.Z.); fs19900520@163.com (S.F.); gyyshiji@126.com (Y.G.)

^2^ State Key Laboratory of Genetic Engineering, School of Life Sciences, Fudan University, Shanghai, 200438, China; Department of Epidemiology, School of Public Health; zhiliangfu@outlook.com (Z.F.); baozi7586@163.com (X.B.)

^3^ Department of Pathology, Shanghai Skin Disease Hospital, Tongji University School of Medicine, Shanghai, 200443, China; wufei_2000@163.com (F.W.)

**^†^** These authors contributed equally to this work.

***** Correspondence: lyqdoctor@163.com (Y.L.); Tel.: +86-021-3680-3100 (Y.L.); wangxiuli_1400023@tongji.edu.cn (X.W.); Tel.: +86-021-3680-3107 (X.W.); sibozhu@fudan.edu.cn (S.Z.); Tel.: +86-021-6410-0038 (S.Z.)

**
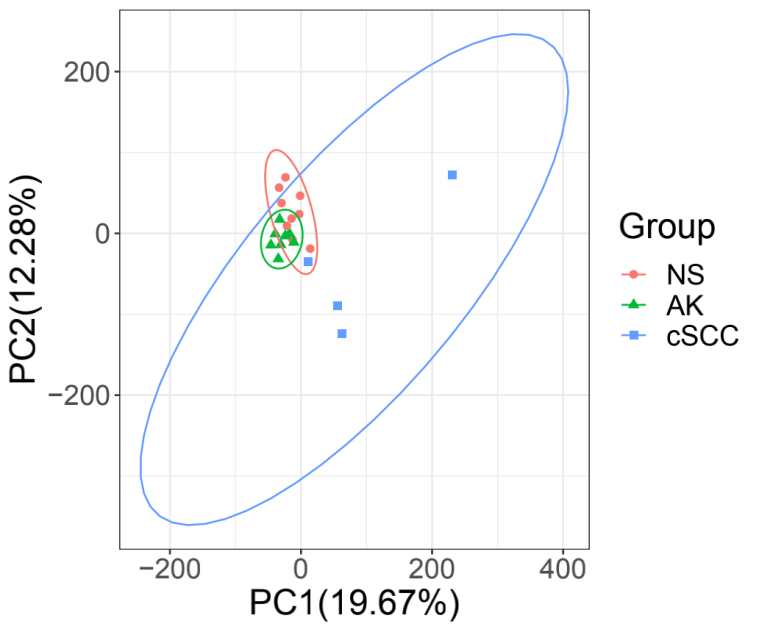
**

**Figure S1.** The PCA diagram among all samples. The PCA diagram shows that the NS and AK group is not distinctively different, while cSCC scattered showing transcriptomic difference over the NS and AK groups.


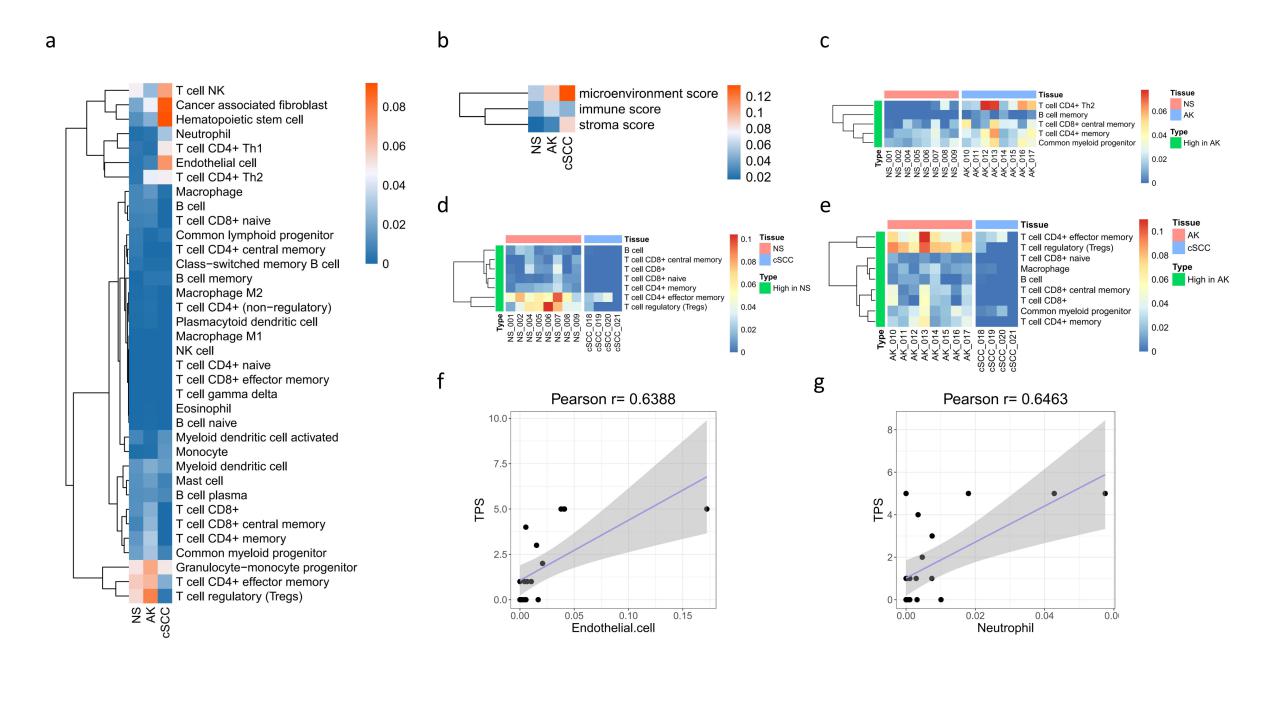


**Figure S2.** RNA deconvolution analyses revealed dynamic change of intratumoral immune and stromal environment. (**a**, **b**, **c**, **d**, **e**) RNA-seq deconvolution to decipher tumor microenvironment. (**f**, **g**) The regression analysis of TPS and microenvironment cell subsets


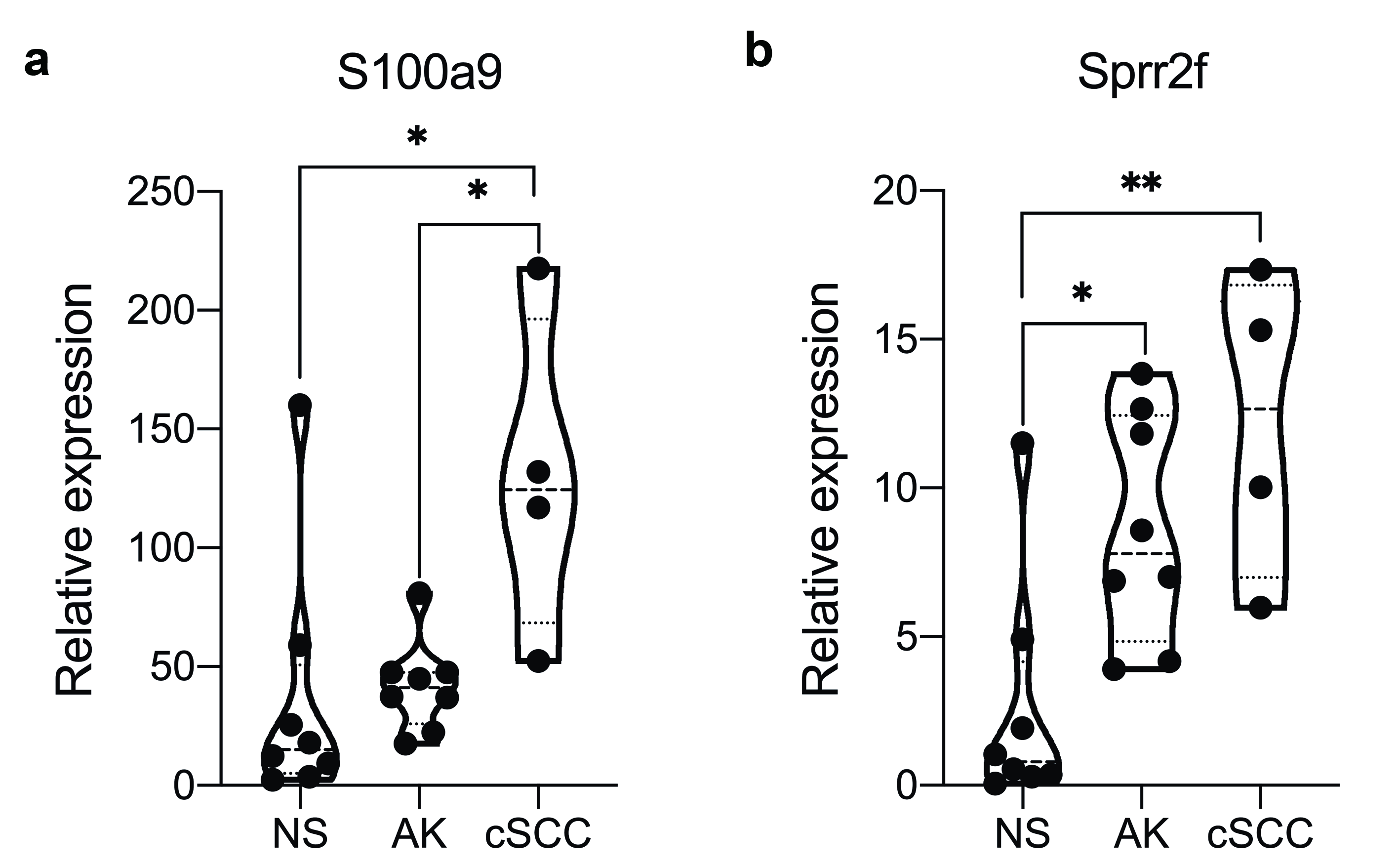


**Figure S3.** Relative expression of S100a9 and Sprr2f. (**a**) S100a9. (**b**) Sprr2f.


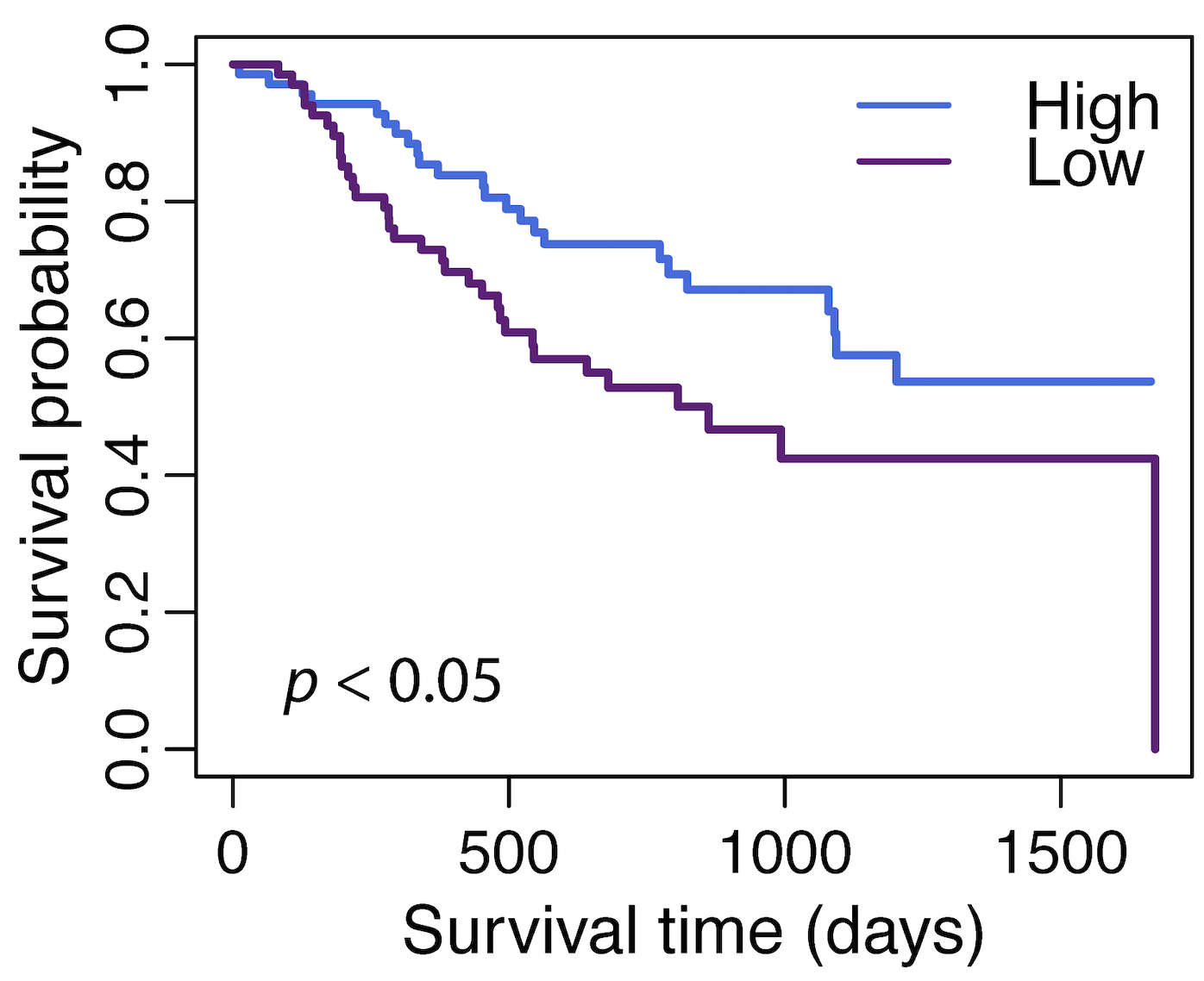


**Figure S4**. Survival time of HNSCC patients can be predicted by Rorα expression. Blue line and purple represent Roar with high and low expression level, respectively


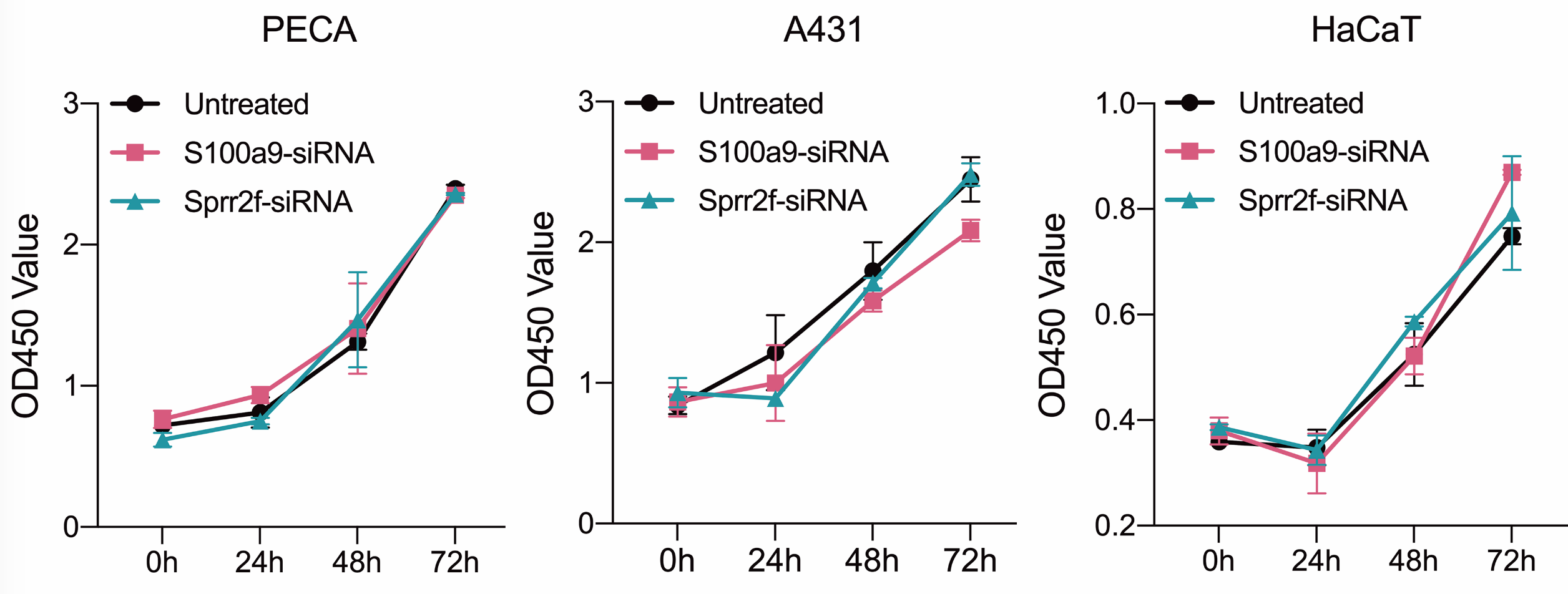


**Figure S5.** CCK8 experiment results in PECA, A431 and HaCaT cell lines. Results showed that siRNA interference with S100a9 and Sprr2f could not promote the proliferation of keratinocytes and skin cancer cells.

**
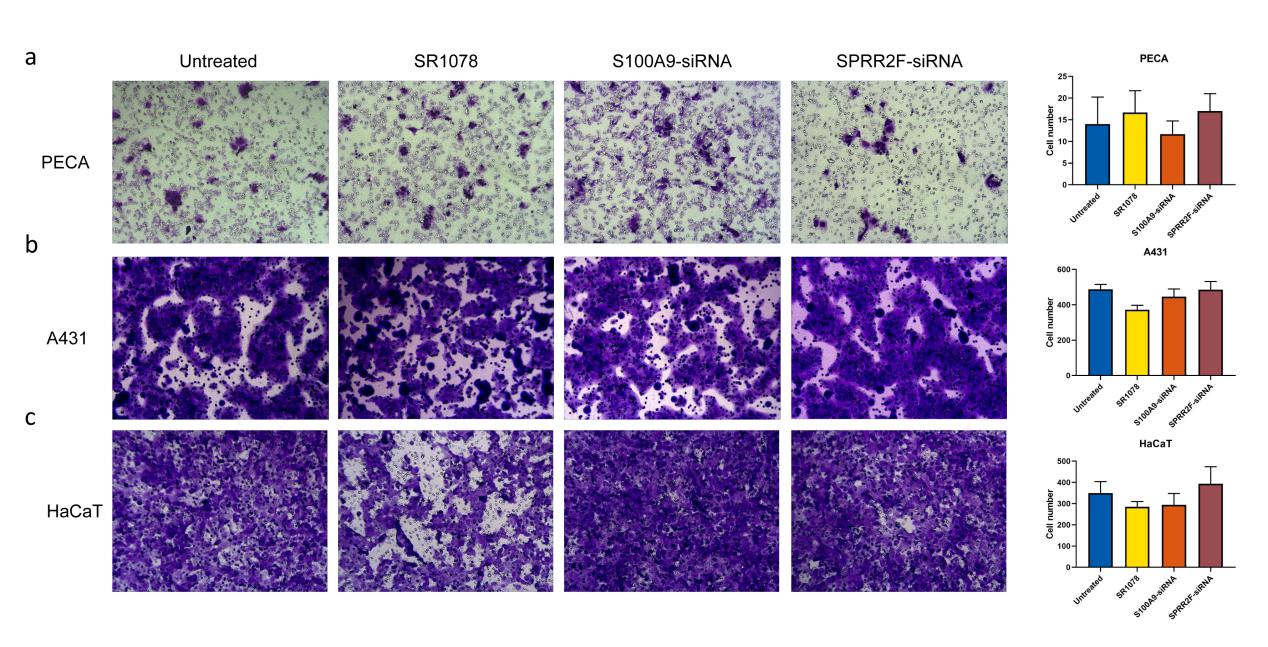
**

**Figure S6.** Transwell experiment results in PECA, A431 and HaCaT cell lines. Results showed that the expression changes of three genes have no effect on the invasion ability.


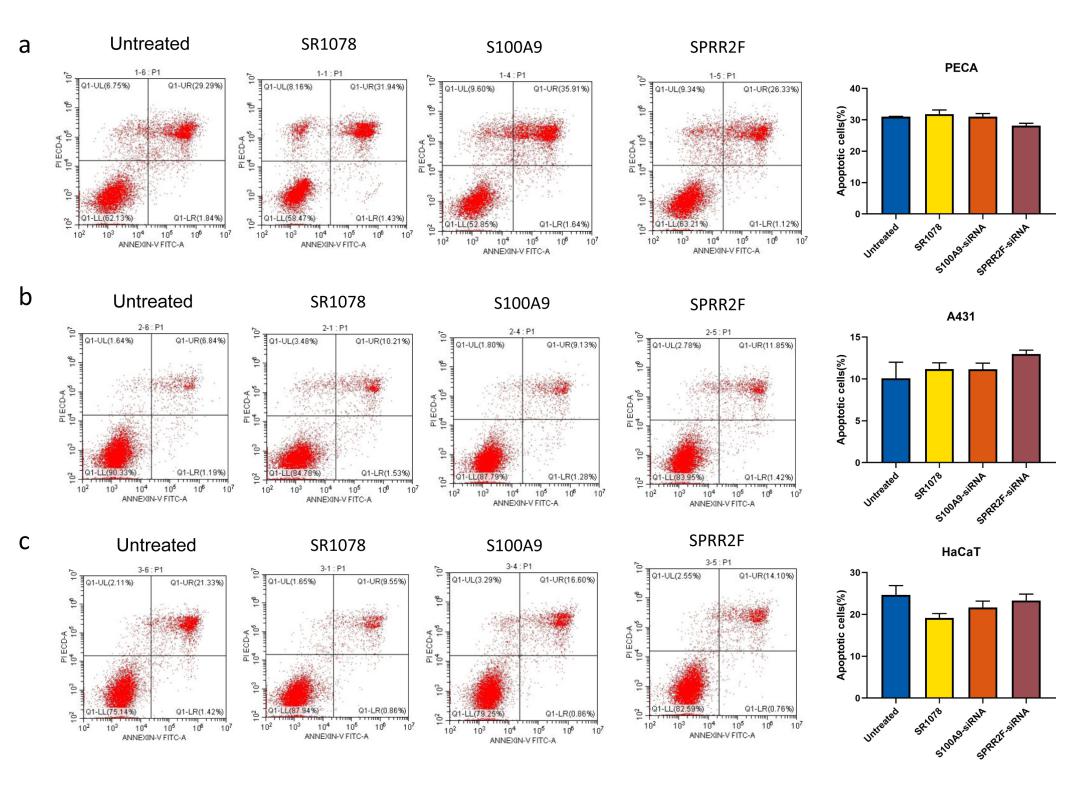


**Figure S7.** The use of flow cytometry technology found that the changes in the expression of three genes did not affect the apoptosis of keratinocytes and skin cancer cells.
